# *Xylella fastidiosa* causes transcriptional shifts that precede tylose formation and starch depletion in xylem

**DOI:** 10.1111/mpp.13016

**Published:** 2020-11-20

**Authors:** Brian Ingel, Clarissa Reyes, Mélanie Massonnet, Bailey Boudreau, Yuling Sun, Qiang Sun, Andrew J. McElrone, Dario Cantu, M. Caroline Roper

**Affiliations:** ^1^ Department of Microbiology and Plant Pathology University of California Riverside California USA; ^2^ United States Department of Agriculture Agricultural Research Service Davis California USA; ^3^ Department of Viticulture and Enology University of California Davis California USA; ^4^ Department of Biology University of Wisconsin Stevens Point Wisconsin USA; ^5^ Wellesley College Wellesley Massachusetts USA; ^6^ Present address: Department of Plant Pathology University of California Davis California USA

**Keywords:** statch, tylose, *Vitis vinifera*, *Xylella fastidiosa*, xylem, xylem ray parenchyma

## Abstract

Pierce's disease (PD) in grapevine (*Vitis vinifera*) is caused by the bacterial pathogen *Xylella fastidiosa*. *X. fastidiosa* is limited to the xylem tissue and following infection induces extensive plant‐derived xylem blockages, primarily in the form of tyloses. Tylose‐mediated vessel occlusions are a hallmark of PD, particularly in susceptible *V. vinifera*. We temporally monitored tylose development over the course of the disease to link symptom severity to the level of tylose occlusion and the presence/absence of the bacterial pathogen at fine‐scale resolution. The majority of vessels containing tyloses were devoid of bacterial cells, indicating that direct, localized perception of *X. fastidiosa* was not a primary cause of tylose formation. In addition, we used X‐ray computed microtomography and machine‐learning to determine that *X. fastidiosa* induces significant starch depletion in xylem ray parenchyma cells. This suggests that a signalling mechanism emanating from the vessels colonized by bacteria enables a systemic response to *X. fastidiosa* infection. To understand the transcriptional changes underlying these phenotypes, we integrated global transcriptomics into the phenotypes we tracked over the disease spectrum. Differential gene expression analysis revealed that considerable transcriptomic reprogramming occurred during early PD before symptom appearance. Specifically, we determined that many genes associated with tylose formation (ethylene signalling and cell wall biogenesis) and drought stress were up‐regulated during both Phase I and Phase II of PD. On the contrary, several genes related to photosynthesis and carbon fixation were down‐regulated during both phases. These responses correlate with significant starch depletion observed in ray cells and tylose synthesis in vessels.

## Graphical abstract

During Pierce's disease, *Xylella fastidiosa* triggers transcriptional changes in *Vitis vinifera* grapevines and induces major physiological responses, including tylose formation and starch depletion.

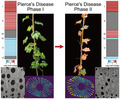

## 1 INTRODUCTION

*Xylella fastidiosa* is a gram‐negative proteobacterium that colonizes and is limited to the xylem of its plant hosts. *X. fastidiosa* is the causal agent of many significant agricultural plant diseases such as Pierce's disease (PD) of grapevine (*Vitis vinifera*), citrus variegated chlorosis, coffee leaf scorch, and, most recently, olive quick decline syndrome (Chang et al., 1993; Davis et al., 1978; Li et al., 2001; Saponari et al., 2013). The xylem is composed of many interconnected vessels of finite, variable length that are connected by pit pairs at which adjacent vessels are separated by pit membranes (Brett & Waldron, 1996; Esau, 1977; Thorne et al., 2006b; Tyree & Zimmermann, 2002). Pit membranes are porous structures made of primary plant cell wall material that allow the passage of water and small solutes, regulate the cavitation threshold, and prevent the free movement of air embolisms and pathogens (Choat et al., 2008; Sperry et al., 1991; Stevenson et al., 2004; Tyree & Zimmermann, 2002). To systemically colonize the xylem, *X. fastidiosa* enzymatically degrades xylem pit membranes to move between vessels (Ingel et al., 2019; Perez‐Donoso et al., 2010; Roper et al., 2007; Sun et al., 2011). Movement between hosts is facilitated by xylem‐feeding hemipteran leafhopper insects, such as the glassy‐winged sharpshooter (*Homalodisca vitripennis*) (Hill & Purcell, 1995). External symptoms associated with PD include leaf scorching, irregular periderm development, berry desiccation, irregular leaf abscission, general stunting, and vine death (Rapicavoli et al., 2018a). The leaf scorching symptom associated with *X. fastidiosa*‐related diseases shares some similarity to leaf scorching observed in grapevines suffering from drought stress (i.e., chlorosis and scorching/necrosis). However, there are distinct differences, especially in how these symptoms manifest (McElrone et al., 2001, 2003; Thorne et al., 2006a). The most apparent difference is that leaves with PD do not wilt as they would during acute or chronic drought stress. The internal symptoms associated with PD include occlusions within the xylem that are composed of plant‐derived pectin gels, crystals, and tyloses, with the latter being the predominant form of vessel occlusion observed in PD‐infected grapevines (Sun et al., 2013).

Tyloses develop, in part, in response to biotic stress, such as pathogen ingress. Their role in host defence responses is to slow or prevent pathogen movement within the xylem, and the success of this defence is dependent on the rate of tylose formation relative to the rate of pathogen movement (Bonsen & Kučera, 1990; Del Rio et al., 2001). Specifically, if tyloses are produced too slowly they are not effective at blocking the pathogen's movement. However, the overproduction of tyloses in response to biotic stress can cause a detrimental reduction in hydraulic conductivity within the xylem (Collins et al., 2009; McElrone et al., 2010). Thus, a fine balance of the amount and timing of tylose production determines the success of whether tyloses can halt pathogen ingress. Indeed, overproduction of tyloses in PD‐impacted grapevines leads to a significant loss in hydraulic conductivity, but is not effective at preventing *X. fastidiosa* from moving systemically in susceptible *V. vinifera* (Deyett et al., 2019; Sun et al., 2013).

Water stress caused by tyloses can have a significant effect on major physiological processes that subsequently affect carbon uptake and utilization. Specifically, plants close their stomata to reduce the rate of transpiration to mitigate water loss and the increased risk of cavitation, but the consequence of this is a significant reduction in photosynthesis and plant growth (Pinheiro & Chaves, 2010). Despite the reduction in photosynthesis, the near cessation of plant growth causes carbon to be stored as starch in the stem xylem rays (Estiarte & Peñuelas, 1999; Hummel et al., 2010). Under acute water stress conditions, plants can use this accumulated starch to support essential processes such as respiration, metabolism, and defence (McDowell, 2011). However, prolonged water stress caused by an increasing number of vessel blockages can result in a prolonged reduction in photosynthesis (Brodribb & Holbrook, 2006; Hölttä et al., 2009). Without an adequate supply of carbon from the leaves over time, starch reserves begin to deplete as the amount of carbon required for survival becomes greater than the amount of carbon available (Gibon et al., 2009; McDowell, 2011). This phenomenon is known as carbon starvation and, when combined with hydraulic failure from chronic water stress, it can cause leaf shedding, disruption of vascular transport, and plant death (Brodribb & Holbrook, 2006; Sevanto, 2014; Tyree et al., 1993; Tyree & Sperry, 1989). This series of events can be further exacerbated by pathogen proliferation as carbon‐costly biotic stress responses decline due to carbon deficit, ultimately hastening plant death (Guérard et al., 2007; McDowell, 2011; Poorter & Villar, 1997).

Previous foundational studies in the PD pathosystem indicate that external symptom development is tightly correlated with internal tylose production (Sun et al., 2013). However, little was known about the timing of initiation and the pattern of tylose occlusion within a grapevine over the course of disease progression. A primary goal of this study was to explore the temporal and spatial patterns of tylose production over the course of the disease in a susceptible grapevine species, *V. vinifera*, and how that relates to the distribution of the pathogen in xylem vessels. Our study revealed that chronic tylose‐induced/derived vessel blockage associated with PD is coupled with starch depletion in the xylem parenchyma cells. Furthermore, we determined that major transcriptional reprogramming associated with both tylose initiation and distribution of carbon stores preceded any visible internal or external symptom development.

## 2 RESULTS

### 2.1 The percentage of tylose‐occluded vessels increases from Phase I to Phase II of PD

**FIGURE 1:**
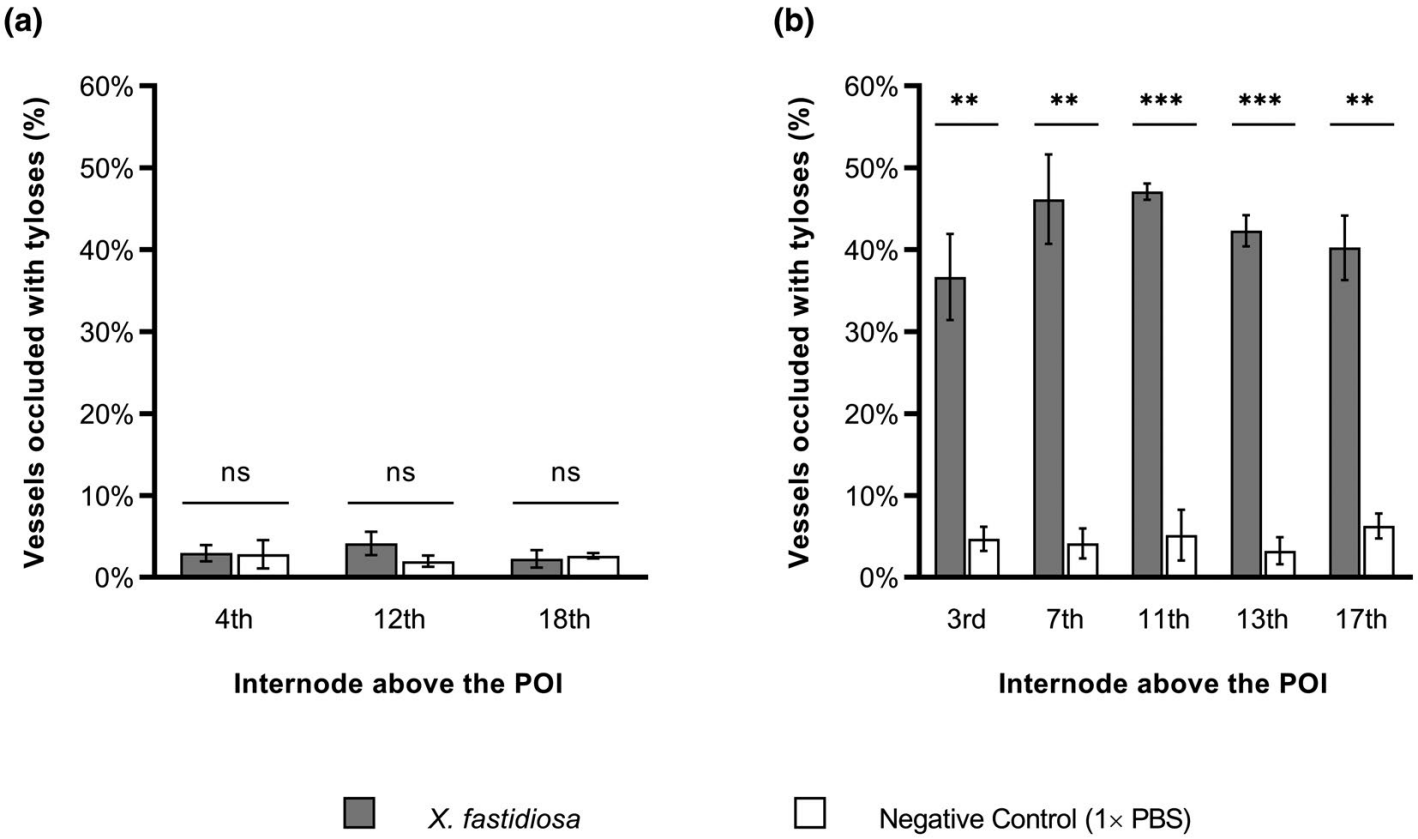
Tylose production increases between Phase I and Phase II of Pierce's disease. Quantitative comparison of tyloses based on percentage of occluded vessels in *Vitis vinifera* 'Cabernet Sauvignon' vines inoculated with *Xylella fastidiosa* (Temecula 1; grey) or phosphate‐buffered saline (PBS) (negative control; white) during Phase I (a) and Phase II (b) of Pierce's disease. Each datum point is presented as mean based on four samples from each of three vines. POI, point of inoculation. Bars represent the standard error of the mean. Statistical comparisons at each internode were performed using Welch's two‐sample *t* test with a significance cut‐off of 0.05. Asterisks indicate the level of significance: *p < .05, **p < .01, ***p < .001

**FIGURE 2:**
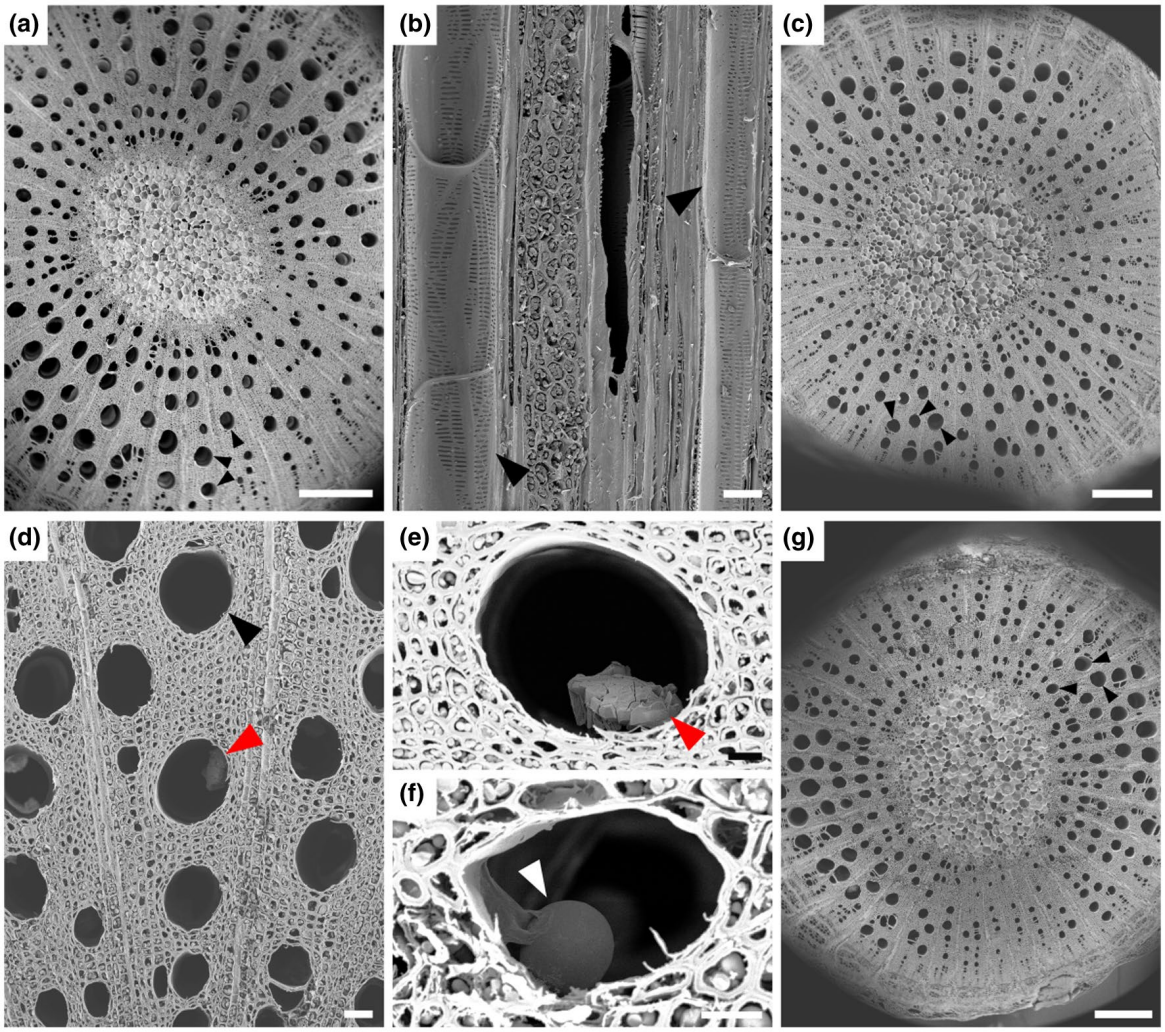
Phase I of Pierce's disease: absence of vascular occlusion in both *Xylella fastidiosa*‐ and phosphate‐buffered saline (PBS)‐inoculated *Vitis vinifera* 'Cabernet Sauvignon' vines. Vascular occlusion during Phase I of Pierce's disease in *V. vinifera* 'Cabernet Sauvignon' vines inoculated with PBS (a, b) or *X. fastidiosa* (c–g), respectively. (a, c–g) Transverse section of stem. (b) Tangential longitudinal section of stem secondary xylem. (a–f) Xylem tissue from the fourth internode counting upward from the point of inoculation (POI) (the same below). (a) Vessels appear as open pores without visible occlusions (black arrows). (b) Several longitudinally transected vessels are free of occlusions (black arrows), showing no tyloses or crystals inside. (c) Xylem in a stem does not contain vessels with obvious occlusion (black arrows). (d) Most vessels are open with a crystal in one vessel (red arrow). (e) A small crystal (red arrow) in a vessel. (f) A small tylose (white arrow) in a vessel lumen. (g) Xylem tissue from the 12th internode showing most vessels are free of tyloses or crystals (black arrows). Scale bar is 500 µm in (a), (c), and (g), 50 µm in (b) and (d), and 20 µm in (e) and (f)

**FIGURE 3:**
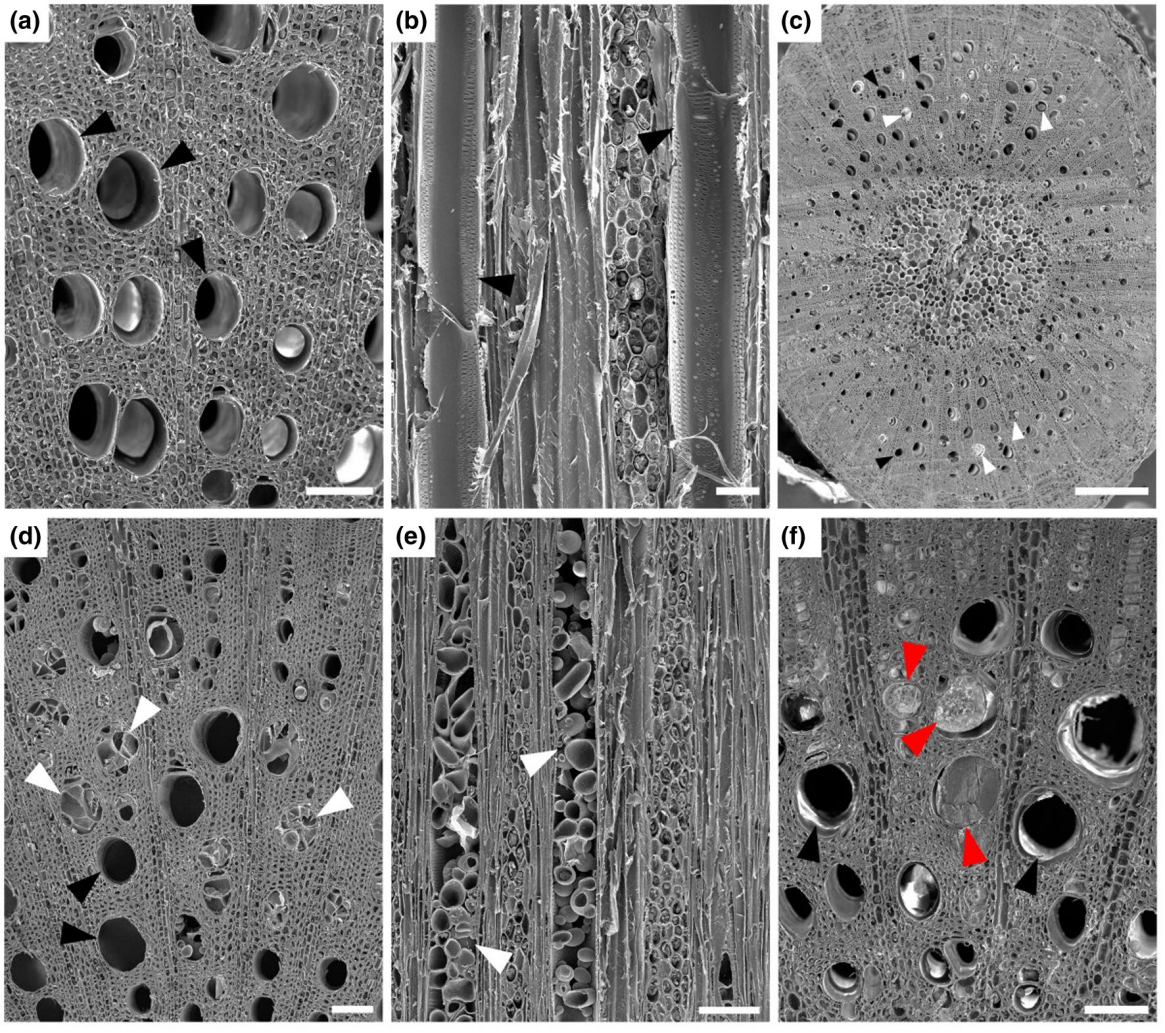
Phase II of Pierce's disease: Vascular occlusion in *Xylella fastidiosa*‐inoculated *Vitis vinifera* 'Cabernet Sauvignon' vines. Vascular occlusion during Phase II of Pierce's disease in *V. vinifera* 'Cabernet Sauvignon' vines inoculated with phosphate‐buffered saline (PBS) (a, b) or *X. fastidiosa* (c–f), respectively. (a), (c), (d), and (f) Transverse section of stem. (b) and (e) Tangential longitudinal section of stem xylem. (a) and (b) Xylem tissues from the third internode from the POI. (a) Vessels are open without occlusions (black arrows) in the transverse section. (b) Two longitudinally transected vessels are free of tyloses (black arrows). (c) Xylem tissue from the third internode from the POI. Both open (black arrows) and occluded vessels (white arrows) are present and occluded vessels occurred as patches dispersed among open vessels. (d) Secondary xylem tissue, showing many vessels completely or partially occluded with tyloses (white arrows), while other vessels remain open (black arrows). (e) Two longitudinally transected vessels filled with tyloses along their lengths (white arrows). (f) Xylem tissue from the 11th internode from the POI, showing that some vessels are fully occluded (red arrows), while other vessels remain open (black arrows). Scale bar is 50 µm in (a) and (b), 100 µm in (d–f), and 500 µm in (c)

We used scanning electron microscopy (SEM) to determine tylose formation, the percentage of occluded vessels, and *X. fastidiosa* distribution above the point of inoculation (POI) during Phase I and Phase II of PD symptom progression. PD symptoms were rated on a weekly basis using the 0–5 PD rating scale established by Guilhabert and Kirkpatrick (2005), where 0 = a healthy vine, 1 = one or two leaves with scorching at the margins, 2 = two or three leaves with more developed scorching, 3 = all leaves with some scorching and a few matchstick petioles, 4 = all leaves with heavy scorching and many matchstick petioles, and 5 = a dead or dying vine. The disease ratings were then further parsed into three disease phases (Figure S1): Phase I (PD symptom score = 1–2), Phase II (PD symptom score = 2–3), and Phase III (PD symptom score = 3–4). Phase I, II, and III plants were sampled at 11, 13, and 14 weeks postinoculation, respectively. During Phase I, only 3% of the vessels in *X. fastidiosa*‐inoculated vines have tyloses or crystalline structures at the fourth internode above the POI, which is similar to phosphate‐buffered saline (PBS)‐inoculated negative control vines at the fourth internode above the POI (*p* = .95), and they are free of other vascular occlusions (Figures 1a and 2a–f). Similarly, extremely low percentages (2%–4%) of vessels associated with tyloses were also found in the negative control vines as well as *X. fastidiosa*‐inoculated vines at the 12th or 18th internode above the POI (*p* = .24 and .76, respectively) (Figures 1a and 2g). Because there were no significant differences in the percentages of vessels containing tyloses between the pathogen and mock‐inoculated vines during Phase I of the disease, we speculate that the tyloses we did observe in both treatments were derived from other unknown intrinsic factors associated with plant development. During Phase II of PD symptom progression, 36.7%–47.1% of vessels contained tyloses in the *X. fastidiosa*‐inoculated vines, while 3.2%–6.3% of vessels contained tyloses in the PBS‐inoculated negative control vines at the 3rd (*p* = .0065), 7th (*p* = .0026), 11th (*p* = .0004), 13th (*p* < .0001), and 17th (*p* = .0015) internodes above the POI (Figure 1b). At these internodes above the POI in *X. fastidiosa*‐inoculated vines, open, partially occluded, and fully occluded vessels were observed by SEM, and vessels containing tyloses occurred as random clusters in the secondary xylem (Figure 3c–e). Vessels containing tyloses at these internodes were mostly completely occluded during this phase (Figure 3c,d), whereas the few vessels containing tyloses at the internodes examined during Phase I were mostly partially occluded (Figure 2c,d). Conversely, PBS‐inoculated negative control vines had very low levels of tyloses similar to Phase I at all internodes examined, and vessels were free of other occlusions (Figures 1 and 3a,b).

### 2.2 *X. fastidiosa* cells are randomly and systemically distributed throughout the xylem during Phase II of PD

**FIGURE 4:**
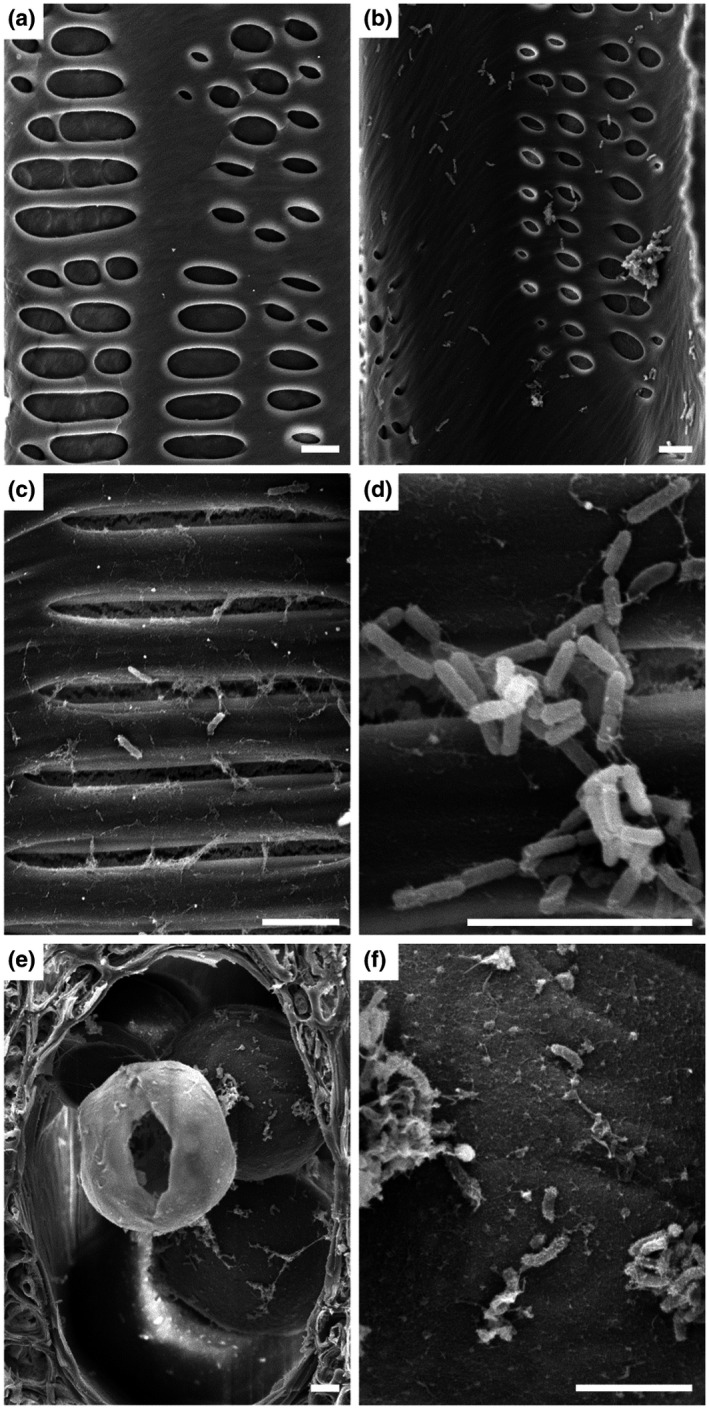
Local and distal presence of *Xylella fastidiosa* in *Vitis vinifera* 'Cabernet Sauvignon' vines during Phase II of Pierce's disease. *X. fastidiosa* distribution during Phase II of Pierce's disease in *V. vinifera* 'Cabernet Sauvignon' vines inoculated with phosphate‐buffered saline (PBS) (a) or *X. fastidiosa* (b–f), respectively. (a–d) A lateral wall of a longitudinally transected vessel. (e, f) Transverse section of a vessel. (a) No *X. fastidiosa* cells in vessels of vines inoculated with PBS. (b) *X. fastidiosa* cells present both individually and in aggregates in a vessel from the 11th internode above the point of inoculation (POI). (c, d) Vessel from the 3rd internode above the POI, showing that *X. fastidiosa* cells are present in small quantities either individually (c) or in small aggregates (d). (e) A vessel from the 17th internode contains both tyloses and *X. fastidiosa* cells. (f) An enlargement of the frame region in (e), showing *X. fastidiosa* cells colonizing the surface of tyloses. Scale bar in all panels is 5 µm

Despite PD symptom development initiating during Phase I, *X. fastidiosa* cells were elusive and not observed in the xylem vessels of infected vines (data not shown). However, by Phase II of PD symptom progression, *X. fastidiosa* cells were readily observable in approximately 10% of the vessels that may or may not contain occlusions in infected vines, and were present in systemic locations throughout these vines (Figure 4). *X. fastidiosa* cells appeared both as individual cells and as small cell aggregates along the lateral walls of the xylem vessels (Figure 4b–d). *X. fastidiosa* cell aggregates large enough to completely occlude a vessel in the secondary xylem were never observed. When *X. fastidiosa* was in a vessel with tyloses, some cells or cell aggregates were observed adhering to a tylose (Figure 4e,f), suggesting that tyloses may provide a desirable surface for *X. fastidiosa* colonization, possibly due to the presence of by‐products of cell wall turnover associated with the developing tylose cell wall (Mohammadi et al., 2012). Furthermore, visualized internodes contained far more tylose‐occluded vessels (Figure 1b) than vessels containing *X. fastidiosa*, indicating that localized bacterial presence is not required for tylose development in many vessels. This suggests that a signal emanates from vessels colonized by *X. fastidiosa* that induces tylose formation in vessels where *X. fastidiosa* is not observed. No *X. fastidiosa* cells were found in any vessels of the PBS‐inoculated negative control vines (Figure 4a).

### 2.3 *X. fastidiosa* infection induces starch depletion in xylem ray parenchyma

**FIGURE 5:**
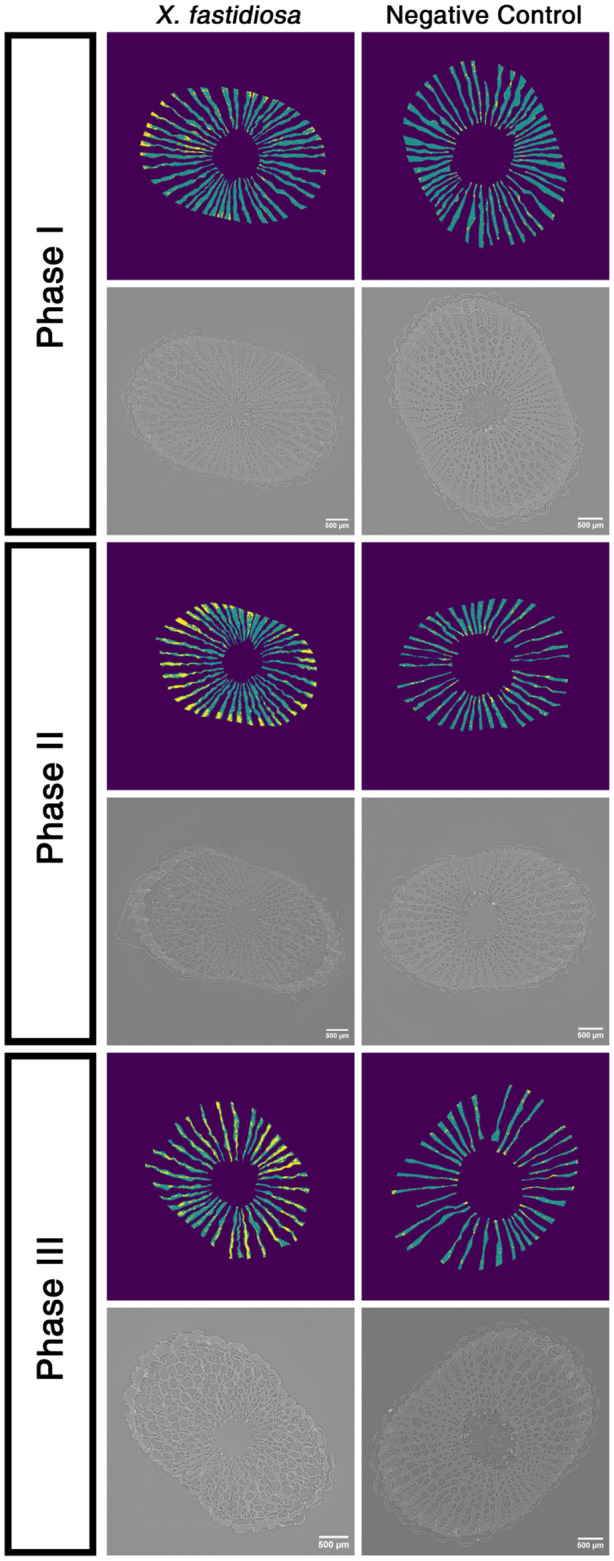
Starch in the xylem ray parenchyma of *Xylella fastidiosa*‐inoculated vines depletes as Pierce's disease symptoms progress. X‐ray computed microtomography (microCT) and a machine‐learning algorithm were used to predict starch depletion at the second internode above the point of inoculation in *X. fastidiosa*‐ and phosphate‐buffered saline (PBS)‐inoculated (negative control) vines during Phase I (top), Phase II (middle), and Phase III (bottom) of Pierce's disease. For each phase, the top row consists of spatial maps depicting the predicted starch‐filled ray parenchyma (blue) and the predicted empty ray parenchyma (yellow). The bottom row for each phase consists of the corresponding reconstructed X‐ray microCT images used with the machine‐learning algorithm to generate the respective spatial maps above

We used high‐resolution X‐ray computed microtomography (microCT) to visualize and quantify the starch content in ray parenchyma cells located between xylem vessel/fibre sectors. We applied a machine‐learning algorithm developed by Earles et al. (2018) to the images to identify starch‐depleted sections of the ray parenchyma during each phase of PD (Figure 5). In Phase I, the ray parenchyma cells in *X. fastidiosa*‐inoculated vines were nearly all filled with starch, similar to the ray parenchyma cells in PBS‐inoculated negative control vines. Measurable starch depletion in the xylem ray parenchyma cells was apparent as vines shifted from Phase I to Phase II of disease. During Phase III, the starch levels were markedly depleted in *X. fastidiosa*‐inoculated vines, indicating a stark decrease from Phase II starch levels. The ray parenchyma cells remained filled with starch in PBS‐inoculated negative control vines throughout the study.

**FIGURE 6:**
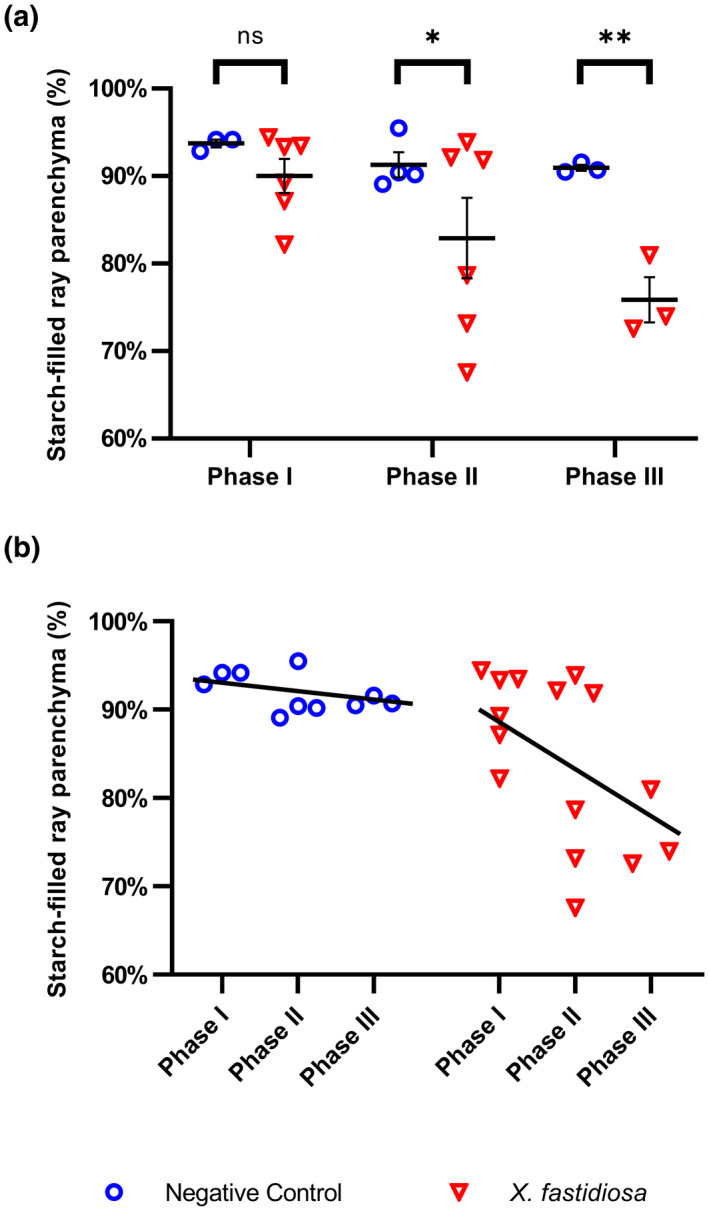
Starch depletion in xylem ray parenchyma cells during different phases of Pierce's disease. Percentage of starch‐filled xylem ray parenchyma was calculated by a machine learning algorithm developed by Earles et al. (2018) during each phase of Pierce's disease in phosphate‐buffered saline (PBS)‐inoculated negative control vines (blue circles) or *Xylella fastidiosa*‐inoculated vines (red triangles). (a) Percentage of starch‐filled xylem ray parenchyma of each individual sample analysed in each of the three disease phases. Black horizontal bars at each phase represent the mean percentage of starch‐filled ray parenchyma of at least three vines and error bars represent the standard error of the mean. Comparisons at each phase were performed using a linear beta regression model, and significance was determined using least squares means with *p* value adjustment via the Tukey–Kramer method. Asterisks indicate the level of significance: *p < .05, **p < .01, ***p < .001. (b) Statistical analysis of the overall depletion of starch from Phase I to Phase III (slope, black line) was also compared using the same model: *X. fastidiosa*‐inoculated vines exhibited significant starch depletion (*p* = .009), while PBS‐inoculated vines did not (*p* = .074)

The algorithm was then used to quantify the percentage of starch‐filled ray parenchyma. During Phase I, both *X. fastidiosa*‐inoculated vines and PBS‐inoculated negative control vines had similar levels of starch (90% filled and 93.7% filled, respectively; *p* = .061; Figure 6a). However, a significant reduction of starch‐filled ray parenchyma was observed from Phase I (90.0% filled) to Phase III (75.9% filled) in *X. fastidiosa*‐inoculated vines (*p* = .009), whereas no such reduction was observed from Phase I (93.7% filled) to Phase III (90.9% filled) in PBS‐inoculated vines (*p* = .074; Figure 6b). Taken together, these results indicate that the marked starch depletion observed occurs as a result of PD and was not due to normal seasonal changes.

### 2.4 Genome‐wide expression profiling indicates differential expression of plant genes related to tylose formation, water stress, and carbon fixation in infected vines

The transcriptomes of *X. fastidiosa*‐ and PBS‐inoculated vines were profiled using RNA sequencing (RNA‐Seq) during Phase I and Phase II of PD symptom progression to capture the vine's response to *X. fastidiosa* during early and mid‐disease. An average of 12.9 ± 1.1 million high‐quality reads were aligned to the grape transcriptome (Table S1). A total of 5,651 differentially expressed genes (DEGs) were detected in response to *X. fastidiosa* infection (*p* ≤ .05), with a higher number of DEGs detected during Phase I (4,636) than in Phase II (2,262) (Figure S3a and Data set S1). Moreover, we found that more genes were up‐regulated rather than down‐regulated during both disease phases. Comparison of the up‐ and down‐regulated genes showed that about half (53%) of the DEGs detected during Phase II were also modulated during Phase I (Figure S3b). These results indicate that an extensive transcriptional reprogramming was initiated in response to *X. fastidiosa* during Phase I even though there was only a low level of visible external symptoms and internal vascular symptoms. Furthermore, an overlap of DEGs during Phase I and Phase II indicates there is commonality among the biological processes involved in the plant response as PD symptoms progress from mild to moderate.

**FIGURE 7:**
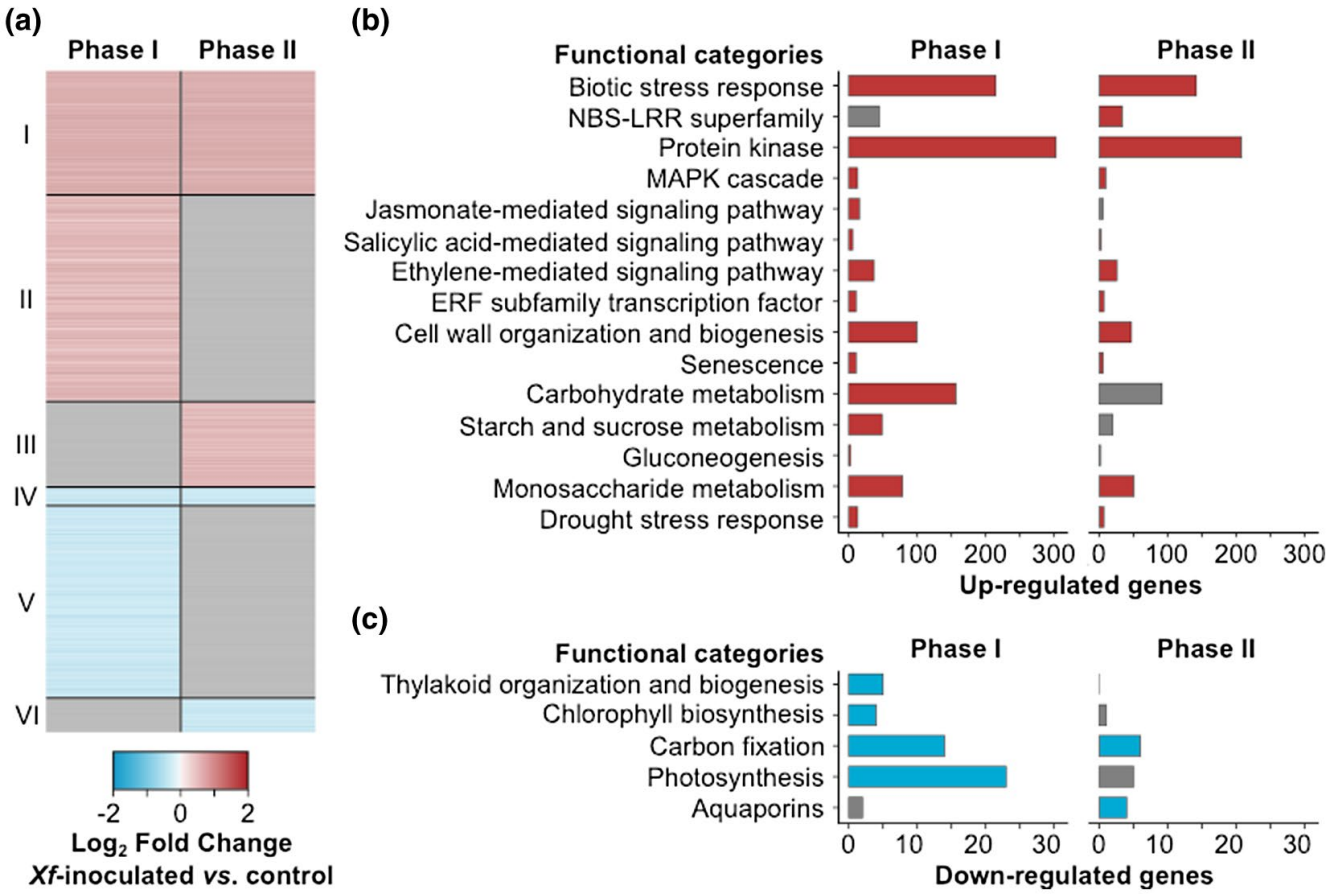
Grapevine transcriptomic response to *Xylella fastidiosa*. (a) Differentially expressed genes (DEGs; *p* ≤ .05) in response to *X. fastidiosa* when compared to phosphate‐buffered saline (PBS) control (c). Genes are classified in six groups (I–VI) based on their expression modulation. The colours of the heat map depict the gene expression fold‐changes (log_2_) between *X. fastidiosa*‐ and PBS‐inoculated vines per DEG. In the absence of significant differential gene expression, log_2_‐transformed fold‐changes are coloured in grey. Enriched grape functional categories (FCTs) (*p* ≤ .05) among genes up‐regulated (red; b) and down‐regulated (blue; c) in response to *X. fastidiosa* infection. Grey bars represent functional categories that are not significantly overrepresented among the DEGs

We classified the DEGs during Phase I and Phase II in *X. fastidiosa*‐inoculated vines into six groups (I–VI) based on their expression modulation in response to *X. fastidiosa* infection (Figure 7a). Overrepresented functional categories among the DEGs at the two phases, as well as among the six groups, were determined by enrichment analysis (Fisher's exact test, *p* ≤ .05; Figure 7b and Data set S2). Group I corresponded to genes that were significantly up‐regulated during both Phase I and Phase II (1,052 genes). The overrepresented functional categories among Group I comprised “Biotic stress response”, “Protein kinase”, “Salicylic acid‐mediated signalling pathway”, and “Drought stress response” (Figure 7b and Data set S2). In addition, the functional category “Cell wall organization and biogenesis” was found enriched among the up‐regulated genes at both Phase I and Phase II (Figure 7b and Data set S2). Induction of this biological process suggests the production and/or expansion of the plant cell wall in response to *X. fastidiosa* infection, possibly to replace the cell wall material damaged by the bacterial cell wall‐degrading enzymes (Ingel et al., 2019; Roper et al., 2007) or to form tyloses (De Micco et al., 2016). Interestingly, a higher number of genes belonging to the functional category “Cell wall organization and biogenesis” were up‐regulated at Phase I (100 genes) compared to Phase II (47 genes; Figure S4), suggesting that the induction of the biological process occurred prior to the formation of tyloses.

Group II consisted of genes that were significantly up‐regulated in response to *X. fastidiosa* infection only during Phase I of the disease. Notably, we found the functional categories “Carbohydrate metabolism” and “Starch and sucrose metabolism” significantly enriched among Group II (Data set S2). These comprised genes associated with carbohydrate, starch, and sucrose metabolism, such as an ADP‐glucose pyrophosphorylase, two sucrose synthases, five glucose‐6‐phosphate/phosphate translocators, a vacuolar invertase, and four alcohol dehydrogenases including *VviADH1* and *VviADH2* (VIT_18s0001g15410 and VIT_04s0044g01110, respectively; Tesnière & Verriès, 2000). The up‐regulation of genes involved in carbon partitioning supports our hypothesis that early *X. fastidiosa* infection is perceived as drought stress and causes plants to subsequently accumulate starch reserves in the xylem parenchyma. The functional category “Ethylene‐mediated signalling pathway” was also detected as significantly enriched among Group II (Data set S2), including a 1‐aminocyclopropane‐1‐carboxylate synthase and three 1‐aminocyclopropane‐1‐carboxylate oxidases. This functional category was also found enriched among the up‐regulated genes at both disease phases (Figure 7b). Ethylene has been previously shown to be linked to tylose production in PD‐infected vines (Perez‐Donoso et al., 2007; Sun et al., 2007). Among the genes up‐regulated only during Phase II (Group III), the functional category “Callose biosynthesis” was detected as significantly overrepresented (Data set S2) with two genes encoding callose synthases, *VviCalS1* and *VviCalS10* (VIT_13s0156g00210 and VIT_17s0000g10010, respectively), previously shown to be involved in grapevine defence against downy mildew (Yu et al., 2016).

Regarding the genes down‐regulated in response to *X. fastidiosa* infection, the functional category “MYB family transcription factor” was significantly overrepresented among the down‐regulated genes at both phases (Group IV; 158 genes). More specifically, we found the gene *VviMYB172* (Wong et al., 2016; VIT_07s0129g01050) encoding a sucrose‐responsive element binding protein, which is potentially involved in the regulation of sucrose transporters expression in grape berry (VviSREBP; GenBank AAY28930; Glissant, 2005). In addition, the functional category “Carbon fixation” was significantly enriched among genes down‐regulated in *X. fastidiosa*‐inoculated vines during both phases (Figure 7c). Drought stress is associated with reduced carbon fixation where stomata close in response to water deficit to prevent hydraulic failure, which significantly reduces the intake of exogenous carbon dioxide (Chaves, 1991; McDowell, 2011). Group V, corresponding to genes that were down‐regulated during Phase I of the disease progression only, was enriched in functional categories related to photosynthetic metabolism, such as “Photosynthesis”, “Chlorophyll biosynthesis”, and “Thylakoid organization and biogenesis” (Figure 7c and Data set S2). This suggests that as a result of *X. fastidiosa* infection, the plant is slowing down photosynthesis during PD symptom onset. Finally, functional categories related to aquaporins were significantly overrepresented among genes down‐regulated in *X. fastidiosa*‐inoculated vines only during Phase II (Group VI; Data set S2). Aquaporins regulate cellular water homeostasis, and differential expression of genes encoding these proteins occurs during drought stress (Afzal et al., 2016).

## 3 DISCUSSION

Because effective tylose production is a determinant in susceptibility or resistance to PD in grapevines, we sought to better understand the dynamics of tylose development across the PD disease spectrum in susceptible *V. vinifera*. Sparse, nascent tylose production was observed during Phase I of the disease that was not discernible from tylose production in mock‐inoculated vines. However, when grapevines transitioned from Phase I to Phase II, tylose production dramatically increased, with nearly 50% of vessels becoming either partially or fully occluded with tyloses. Interestingly, in Phase I and prior to detectable numbers of bacteria or substantial manifestation of tyloses, major transcriptional reprogramming in biological processes necessary for tylose formation was initiated. Namely, several genes associated with the ethylene biosynthesis pathway and plant cell wall biogenesis were significantly up‐regulated during Phase I. This transcriptional reprogramming in stem tissue suggests that ethylene production occurs during early disease onset, well in advance of notable symptom development, and probably plays a role in initiating a signal that emanates beyond the infected vessels. This corroborates a previous transcriptomics study performed on petiole tissue indicating that gene pathways related to ethylene production were up‐regulated as early as 8 hr after *X. fastidiosa* infection in both local and systemic locations in the vines (Rapicavoli et al., 2018b). Grapevine leaves with PD symptoms produce more ethylene than their healthy counterparts (Perez‐Donoso et al., 2007). Similarly, ethylene production in xylem tissue was associated with rapid tylose production in mature walnuts suffering from apoplexy that results in complete hydraulic failure and tree death within a few weeks (McElrone et al., 2010). We found that up‐regulation of ethylene production and cell wall biogenesis pathways continue during Phase II. Inhibiting the production and signal perception of the wound‐induced ethylene in grapevine may significantly reduce and delay the wound‐induced tylose development (Sun et al., 2007). It is tempting to speculate that suppressing ethylene production early in the development of PD could similarly mitigate the uncontrolled production of tylose development in *X. fastidiosa‐*infected susceptible grapevine species and warrants future experimentation.

Grapevines experience a significant reduction in hydraulic conductivity during Phase III of PD (Sun et al., 2013). The significant increase in tylose‐occluded vessels from Phase I to Phase II of PD, unmitigated by new vessel production, suggests that *X. fastidiosa*‐infected grapevines enter a state of tylose‐mediated prolonged water stress when they shift from a mild to moderate disease status. Prolonged water stress has been linked to depletion of starch reserves and carbon starvation (McDowell, 2011). In control vines and in Phase I *X. fastidiosa‐*inoculated vines, ray parenchyma was replete with starch. However, several genes related to carbohydrate, starch, and sucrose metabolism were up‐regulated in Phase I *X. fastidiosa‐*inoculated vines, indicating that the plant is initiating changes in carbon metabolism as a result of *X. fastidiosa* infection despite the absence of major external disease symptoms. Furthermore, a number of genes associated with drought stress were up‐regulated, which further corroborates previous work that the plant initially perceives *X. fastidiosa* as a form of abiotic water stress as early as 8 hr after the bacteria enter the xylem (Rapicavoli et al., 2018b). Some genes involved in photosynthesis and carbon fixation were down‐regulated in Phase I, suggesting that the plant is beginning to slow down photosynthesis as a result of the perception of bacterial infection. A similar trend has been reported in plants infected by another grapevine trunk pathogen, *Neofusicoccum parvum* (Massonnet et al., 2017), and starch depletion occurs in grapevines infected with the fungal vascular pathogens *Eutypa lata* and *Phaeomoniella chlamydospora* (Pouzoulet et al., 2017; Rolshausen et al., 2008). By Phase II, some carbon fixation genes remained down‐regulated, the number of up‐regulated genes involved in starch accumulation genes decreased, and depletion of starch in the ray parenchyma was apparent. By Phase III, significant starch depletion occurred in the ray parenchyma cells. Pathogen‐induced carbon depletion is not unique to PD, as similar physiological responses have been reported for the vascular fungal pathogen *N. parvum* (Czemmel et al., 2015). However, little is known about xylem‐limited bacterial pathogens regarding starch depletion and whether or not it leads to carbon starvation in planta.

As the percentage of tylose‐occluded vessels increased from Phase I to Phase II, the percentage of vessels containing *X. fastidiosa* cells also increased. However, the percentage of vessels that contained both tyloses and *X. fastidiosa* cells was much lower than the percentage of vessels with tyloses alone, indicating that not all tylose formation is a result of direct, localized interactions between the bacterium and the adjacent plant tissue. Gambetta et al. (2007) also found no clear relationship between bacterial population and PD symptom development, and that high and localized concentrations of *X. fastidiosa* were not necessary for manifestation of leaf‐scorch symptoms. This supports our hypothesis that tylose formation is mediated by a signal probably emanating from the living xylem parenchyma cells adjacent to xylem vessels. This signal(s) remains unknown but the transcriptomics data suggests that ethylene is an important signalling molecule involved in the distal formation of tyloses.

Tyloses that are formed in vessels in the absence of local bacteria could also be caused by air embolisms formed as a result of pathogen‐derived cavitation in the xylem. During drought, reduced water uptake and increased transpiration lower xylem pressure to a point where air is pulled into a water‐filled vessel from adjacent air‐filled spaces, causing the water column to break (resulting in an air embolism) (Hopkins, 1989; Mayr et al., 2014). McElrone et al. (2008) found that petioles of *X. fastidiosa*‐infected oak trees exhibited increased embolism prior to hydraulic failure when vessel occlusion occurred with the onset of scorch symptoms. Some plants produce tyloses in response to air embolisms caused by cavitation resulting from wounding or damage to the vascular tissue to seal affected vessels and divert water to other vessels (De Micco et al., 2016; Zimmermann, 1979). Air embolisms can spread within the xylem if the pressure differential between embolized and water‐filled vessels causes air seeding across the pit membrane or if pit membranes have been ruptured (Choat et al., 2003; Plavcová et al., 2013; Sperry & Tyree, 1988). Perez‐Donoso et al. (2007) observed air embolisms and cavitation using magnetic resonance imaging in grapevines inoculated with *X. fastidiosa* prior to observance of visible external symptoms. Furthermore, asymptomatic regions of infected grapevines experienced significantly more cavitation prior to symptom development relative to healthy control vines, indicating that the cause of cavitation was probably pathogen‐induced (Perez‐Donoso et al., 2007). *X. fastidiosa* produces cell wall‐degrading enzymes, specifically a polygalacturonase and at least one endoglucanase, that act in concert with one another to degrade intervessel pit membranes (Ingel et al., 2019; Perez‐Donoso et al., 2010; Roper et al., 2007; Sun et al., 2011), facilitating the spread of air embolisms as pit membranes are breached. The bulk of the evidence suggests that embolism formation probably precedes vessel occlusion in PD‐infected grapevines similar to other species (e.g., McElrone et al., 2008) even though Sun et al. (2007) showed that tyloses can form in grapevines in the absence of embolism.

In conclusion, the host response to *X. fastidiosa* in susceptible grapevines initiates with major transcriptional reprogramming that results in up‐regulation of the molecular machinery associated with tylose formation and starch utilization, while concomitantly suppressing photosynthesis. Following this, tyloses are extensively produced throughout the xylem, which coincides with depletion of the xylem ray starch. Starch depletion is probably linked to supplying carbohydrates for synthesizing cell wall material for forming tyloses. This starch depletion and down‐regulation of the photosynthetic machinery causes chronic carbon limitations. In addition, hydraulic failure that is also brought on by tyloses later in the disease limits water delivery to the leaves and stomatal closure (and reduces photosynthesis from less carbon dioxide diffusing in). Therefore, thirst and starvation are probably linked and lead the eventual death of the plants that succumb to infection.

## 4 EXPERIMENTAL PROCEDURES

### 4.1 Grapevines and bacterial strains

*V. vinifera* 'Cabernet Sauvignon' grapevines (kindly provided by Foundation Plant Services – UC Davis) were propagated from cuttings in vermiculite trays and transplanted into one‐gallon pots after root and shoot establishment. Vines were pruned to a single shoot, which was tied to a bamboo stake and allowed to grow to 5 ft under greenhouse conditions. Optimal water and nutrients were provided on a consistent basis. *X. fastidiosa* subsp. *fastidiosa* was grown on PD3 agar media at 28 °C for 5 days (Davis et al., 1981). Cells were harvested from PD3 agar plates with PBS and the OD_600 nm_ was adjusted to 0.25 (approximately 10^8^ cfu/ml).

### 4.2 In planta inoculations and PD symptom scoring

Grapevines were mechanically inoculated using a method adapted from Purcell and Sanders (1999). Twenty microlitres of harvested *X. fastidiosa* cells (OD_600 nm_ = 0.25) was placed on the sixth internode of the stem, and the stem was pierced through the droplet using a 20‐gauge needle. The needle was inserted to a depth where it penetrated the xylem, whereby the negative pressure of the xylem facilitated uptake of the inoculum droplet. Grapevines inoculated with PBS served as negative controls and all vines were randomized in the greenhouse. PD symptoms were rated on a weekly basis using the 0–5 PD rating scale established by Guilhabert and Kirkpatrick (2005) where 0 = a healthy vine, 1 = one or two leaves with scorching at the margins, 2 = two or three leaves with more developed scorching, 3 = all leaves with some scorching and a few matchstick petioles, 4 = all leaves with heavy scorching and many matchstick petioles, and 5 = a dead or dying vine. The trial was split up into three disease phases based on the average PD symptom score of the wild‐type‐inoculated vines within each disease phase (Figure S1): Phase I (PD symptom score = 1–2), Phase II (PD symptom score = 2–3), and Phase III (PD symptom score = 3–4). Each disease phase consisted of three biological replicates each containing three technical replicates, totalling nine wild‐type‐inoculated vines and nine negative control vines.

### 4.3 Grapevine tissue sampling

Once the average PD symptom score of the wild‐type‐inoculated vines within a disease phase entered the established range (this occurred at 11, 13, and 14 weeks postinoculation for Phases I, II, and III, respectively), intact stem tissue from those vines and the cognate set negative control vines was sampled for (a) tylose analysis via SEM, (b) starch analysis via X‐ray microCT, and (c) transcriptomic analysis via RNA‐Seq. The internode directly above the POI was excised from the vine and immediately immersed in liquid nitrogen for subsequent RNA extraction and RNA‐Seq analysis. The second internode above the POI was excised was dried at 40 °C for 24 hr in preparation for microCT analysis. The rest of the vine was used for SEM. Details pertaining to disease phase parameters, when samples were acquired, and the number of samples used in each experiment are presented in Table S2.

### 4.4 Scanning electron microscopy

Experimental vines for this study were inoculated with either PBS or wild‐type *X. fastidiosa* (Temecula 1) and were visualized at either Phase I or Phase II of PD symptom development. Three vines from each inoculation type at each PD phase were used to investigate xylem structural features, detect *X. fastidiosa* distribution, and quantify vascular occlusion within stems. For each vine from Phase II (inoculated with either PBS or *X. fastidiosa*), five internode lengths of approximately 2 cm were sampled, from the 3rd, 7th, 11th, 13th, and 17th internodes, counting upwards from the POI, which was considered the internode zero. Sampling occurred in the same way for each vine from Phase I except that samples were collected from the 4th, 12th, and 18th internodes. Samples from Phase I and Phase II were then fixed in PEM (50 mM PIPES, 5 mM EGTA, 5 mM MgSO_4_, pH 6.9) buffer containing 4% paraformaldehyde (PFA) and formalin‐acetic acid‐alcohol (FAA), respectively, for at least 24 hr. Then, multiple 1 mm thick stem disks and segments exposing a radial or tangential surface were trimmed from each fixed sample. Sample trimming was conducted in 50 mM PIPES for the samples fixed with PFA and in 50% ethanol for those fixed with FAA. Trimmed specimens from PFA‐fixed samples were dehydrated through an ethanol series of 10%, 30%, 50%, 70%, 85%, 90%, 95%, 100%, and 100% with a 20 min stay at each step, while those from FAA‐fixed samples started the dehydration process from 70% also with 20 min at each step. Dehydrated specimens were critical‐point‐dried with DCP‐1 (Denton Vacuum, Inc.) or Autosamdri‐931 (Tousimis Research Corp., Inc.) and then sputter‐coated with Au/PD with Desk II (Denton Vacuum, Inc.) or CCU‐010 compact coating unit (Safematic GmbH). Coated specimens were examined and photographed under a scanning electron microscope (Hitachi S3400N, Hitachi Science Systems Ltd) at an accelerating voltage of 8 kV.

Images of transverse sections of stem disks were used to quantify occluded xylem vessels. Stem disks from three vines were analysed for each investigated internode of each inoculation type at either of the two phases. For each stem disk, one‐third of the xylem sector of the whole stem transverse surface was randomly selected to count the total vessel number and the number of vessels with tylose(s) or crystal(s), from which a percentage of vessels with vascular occlusion was calculated and used as a representative of the stem disk's vascular occlusion status. Each datum point of vascular occlusion status was presented as a mean with standard error of three samples/vines.

### 4.5 X‐ray microCT

Dried stem samples from each of the three disease phases were scanned in a 21‐keV synchrotron X‐ray beam using a continuous tomography setting yielding 1,025 two‐dimensional longitudinal images (resolution of 1.27 mm/pixel), which were captured on a complementary metal oxide semiconductor (CMOS) camera (PCO.edge; PCO) at 350 ms exposure time. Acquired raw images were reconstructed into transverse images using a custom software plug‐in for the image‐processing software FIJI (www.fiji.sc; ImageJ) that was developed at the Advance Light Source (Lawrence Berkeley National Laboratory, Beamline 8.3.2). Quantitative analysis was conducted using the middle slice of image stacks reconstructed with standard gridrec Fourier reconstruction to determine starch depletion in ray parenchyma. Starch depletion was quantified using machine‐learning algorithms developed by Earles et al. (2018). At least three representative samples from each treatment within each disease phase were selected for this analysis. Three additional samples from Phase I and Phase II wild‐type‐inoculated vines were added to the analysis to mitigate variability within this treatment during these disease phases.

### 4.6 Statistical analysis of starch depletion

The starch depletion data conformed to a beta distribution with values ranging from 0 to 1 (Figure S2a). A beta regression model was applied to the data using time (phase) and treatment (*X. fastidiosa* and PBS) as independent variables. This model maintained the lowest Akaike information criterion and the Bayesian information criterion scores compared to alternative models, and was considered the model of best fit based on residual analysis (Figure S2b). Post hoc analysis was performed using least square means to generate pairwise comparisons of phase within treatment and comparisons between treatments. *p* values were adjusted using the Tukey–Kramer method. Model generation and post hoc analysis were performed in R (R Core Team, 2019) using betareg v. 3.1‐2 (Cribari‐Neto & Zeileis, 2010) and emmeans v. 1.4.1 (Lenth, 2019).

### 4.7 RNA extraction and library preparation

Stem samples excised from wild‐type‐inoculated vines and negative control vines during Phases I and II were pooled within each of the three biological replicates (three technical replicates per biological replicate; total of nine individual treatment samples per disease phase). The intact stem samples were ground (including the bark) and total RNA was isolated from each pooled biological replicate using a cetyltrimethylammonium bromide‐based extraction protocol as described in Blanco‐Ulate et al. (2013). RNA concentration and purity were measured using a Qubit fluorometer (Thermo Scientific) and a NanoDrop 2000c spectrophotometer (Thermo Scientific), respectively. Libraries were prepared using the Illumina TruSeq RNA sample preparation kit v. 2 (Illumina). Final libraries were evaluated for quantity and quality using the High Sensitivity DNA kit on a Bioanalyzer 2100 (Agilent Technologies).

### 4.8 RNA sequencing and downstream analysis

cDNA libraries were sequenced using an Illumina HiSeq 4000 sequencer (DNA Technologies Core, University of California, Davis, CA, USA) as single‐end 100‐bp reads (Illumina). Illumina reads were trimmed using Trimmomatic v. 0.36 (Bolger et al., 2014) with the options LEADING:3 TRAILING:3 SLIDINGWINDOW:10:20 MINLEN:20. Trimmed single‐end reads were mapped onto the predicted protein‐coding sequences of *V. vinifera* “PN40024” (version V1 from http://genomes.cribi.unipd.it/grape/) using Bowtie2 v. 2.3.4.1 (Langmead & Salzberg, 2012) with parameters ‐q ‐end‐to‐end ‐sensitive ‐no‐unal. Counts of reads mapping uniquely onto the grape reference transcriptome (i.e., with *Q* > 30) were extracted using sam2counts.py v. 0.91 (https://github.com/vsbuffalo/sam2counts). Details of trimming and mapping results are reported in Table S1. The Bioconductor package DESeq2 v. 1.16.1 (Love et al., 2014) was used for read count normalization and for statistical testing of differential gene expression. VitisNet functional annotations were used to assign grape genes to functional categories (Grimplet et al., 2009). Enrichment analyses of grape biological functions were computed in R using the classic Fisher method (*p* ≤ .05).

## AUTHOR CONTRIBUTIONS

M.C.R., Q.S., A.J.M., and D.C. conceived the original research plans and supervised the experiments. B.I. performed the plant inoculation experiments. B.B. performed most of the SEM work. Y.S. performed quantitative analysis of xylem vascular occlusion and part of the SEM work. C.R. performed the starch depletion based on machine‐learning methods. M.M. performed the RNA‐Seq analysis. B.I. wrote the article with contributions of all the authors. M.C.R. supervised and completed the writing. M.C.R. agrees to serve as the author responsible for contact and ensures communication.

## Data Availability

Sequences were deposited at the National Center for Biotechnology Information's Gene Expression Omnibus (GEO) at https://www.ncbi.nlm.nih.gov/geo/ under accession no. GSE152164.
