# Supplementary figures and images for: Xylella fastidiosa causes transcriptional shifts that precede tylose formation and starch depletion in xylem
Source: Mol Plant Pathol. 2020 Nov 20;22(2):175–88. doi: 10.1111/mpp.13016 (PMC7814960; doi:10.1111/mpp.13016)

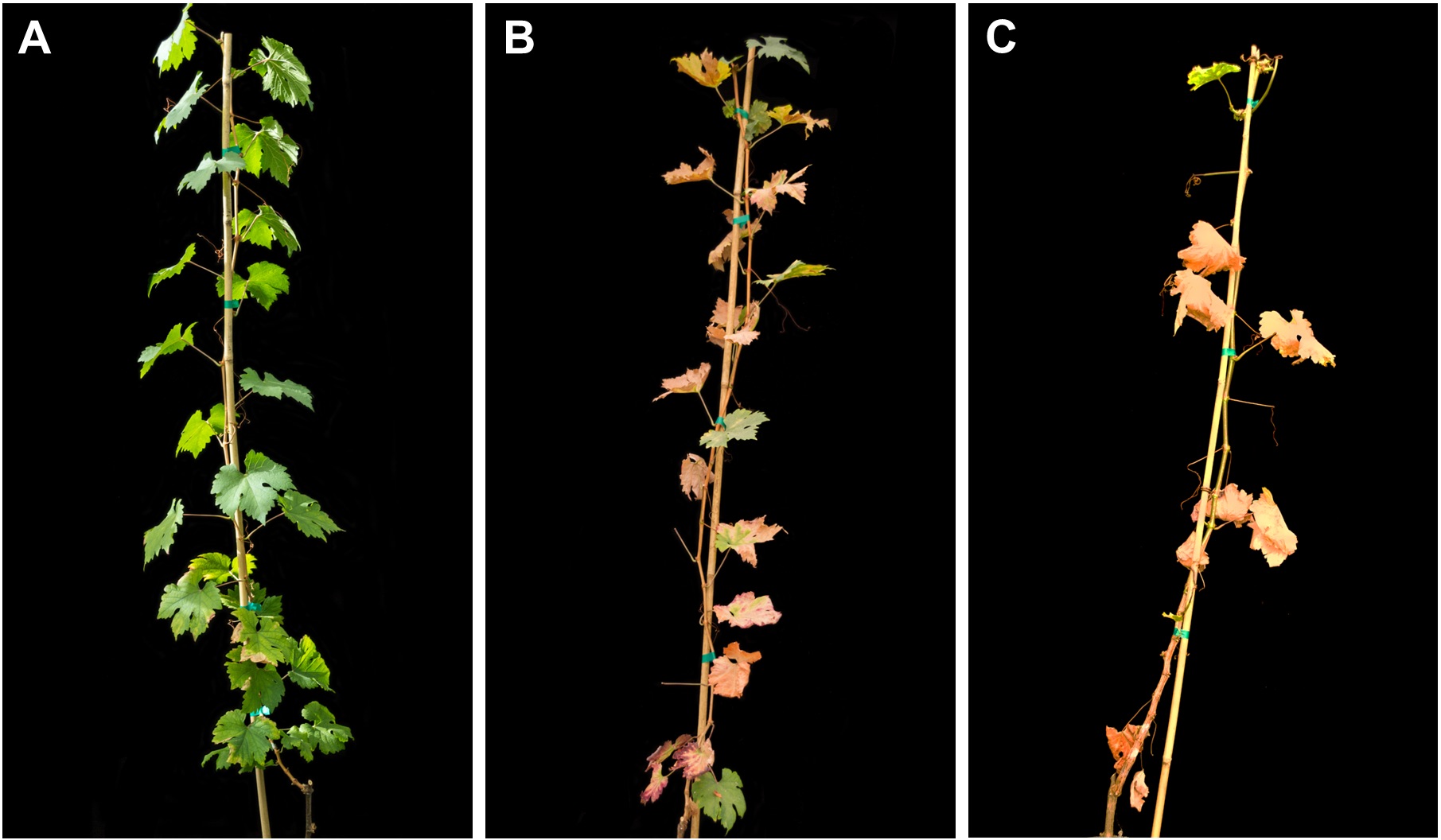

Supplement: Supplementary file 1 [file MPP-22-175-s001.jpg]

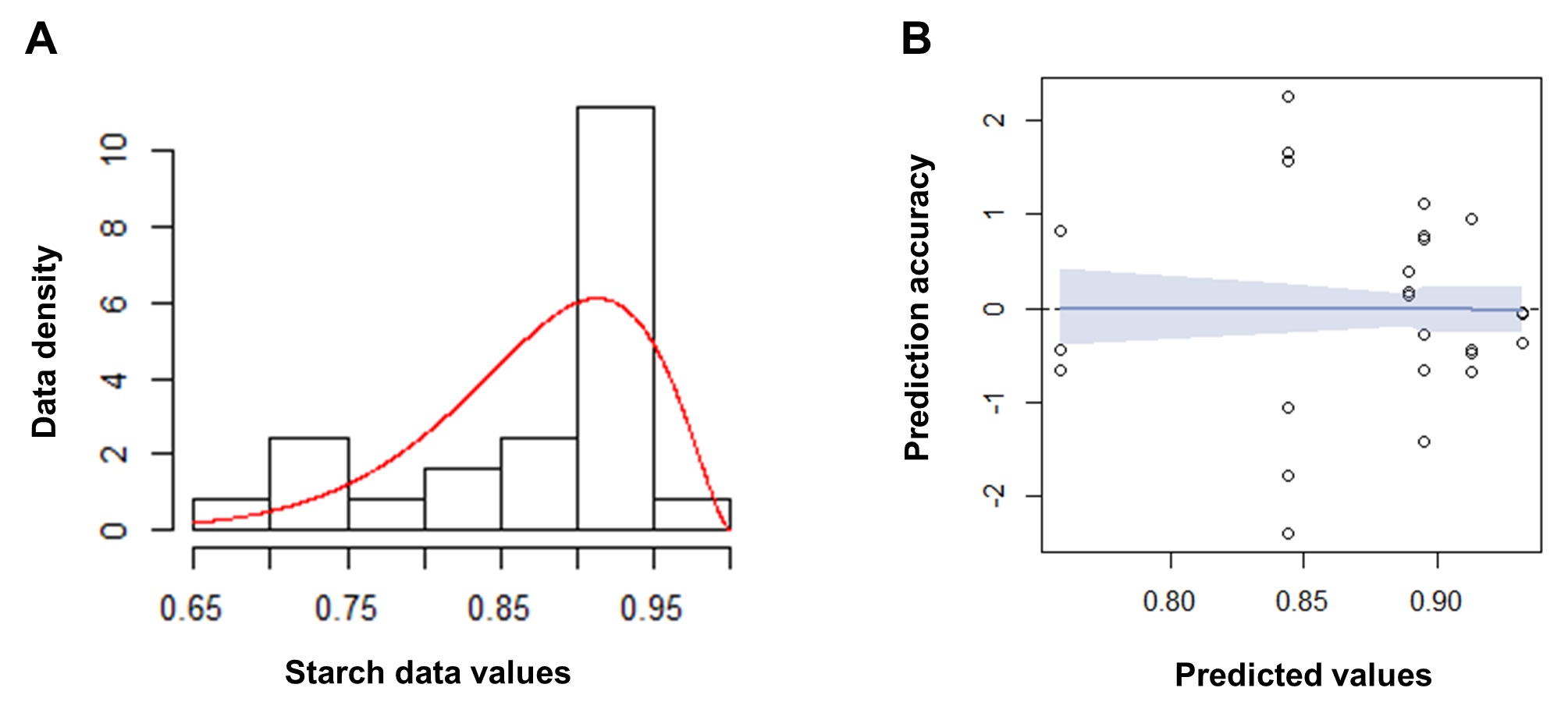

Supplement: Supplementary file 2 [file MPP-22-175-s002.jpg]

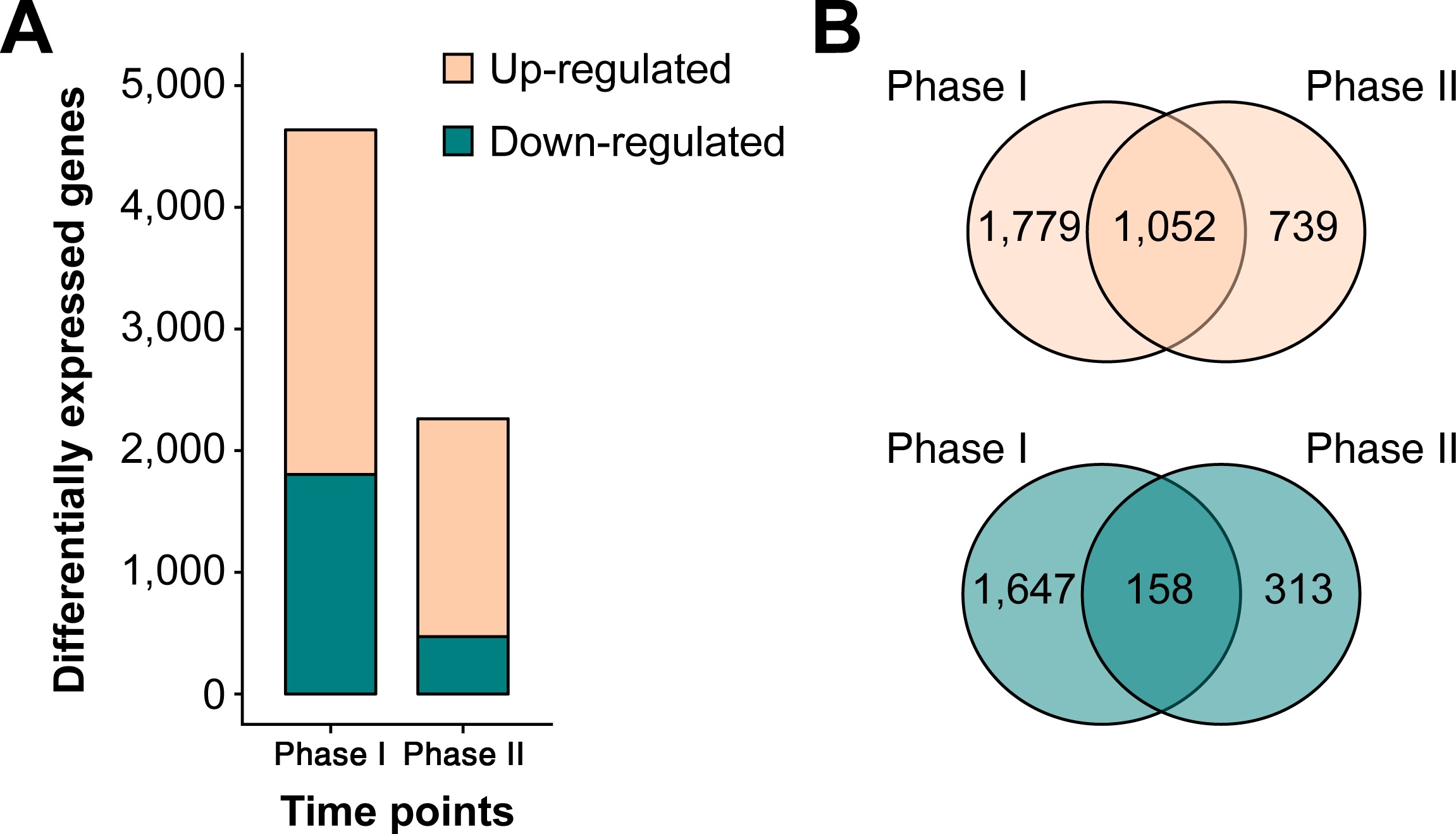

Supplement: Supplementary file 3 [file MPP-22-175-s003.jpg]

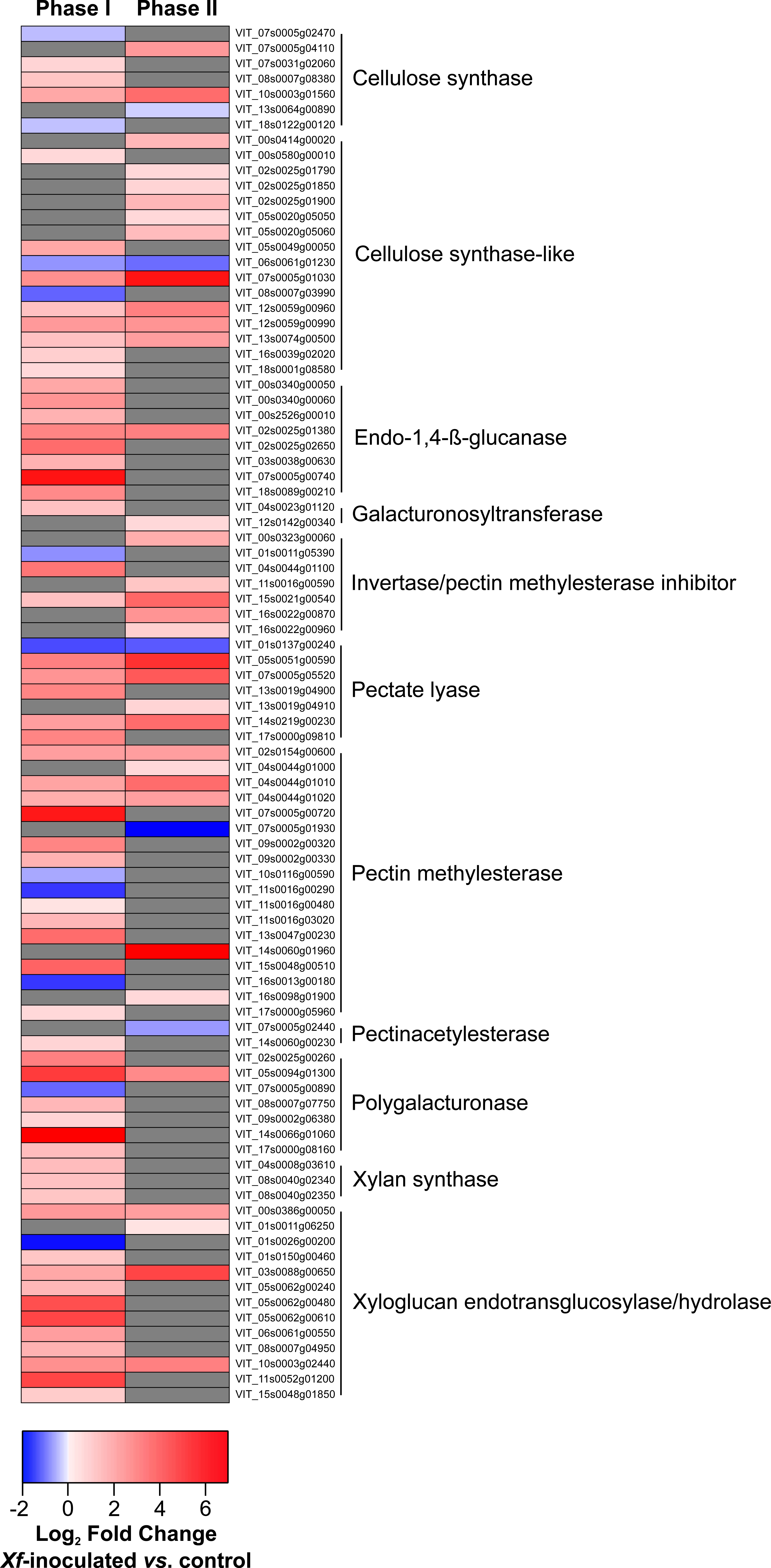

Supplement: Supplementary file 4 [file MPP-22-175-s004.jpg]
